# Unveiling causal links between serum amino acid levels and risk of hepatobiliary neoplasms by Mendelian randomization study

**DOI:** 10.1097/MD.0000000000045491

**Published:** 2025-10-24

**Authors:** Aoqiang Zhai, Li Ren, Yanjie Zhong, Ruiqi Zou, Siqi Yang, Yanwen Jin, Haijie Hu, Fuyu Li

**Affiliations:** a Division of Biliary Tract Surgery, Department of General Surgery, West China Hospital, Sichuan University, Chengdu, Sichuan, China; b Research Center for Biliary Diseases, West China Hospital, Sichuan University, Chengdu, Sichuan, China; c Division of Vascular Surgery, Department of General Surgery, West China Hospital, Sichuan University/West China School of Nursing, Sichuan University, Chengdu, Sichuan, China.

**Keywords:** causal association, hepatobiliary neoplasms, Mendelian randomization, serum amino acids

## Abstract

The causal links between serum amino acids (AAs) and hepatobiliary neoplasms remain unclear. This study aimed to systematically investigate these associations using Mendelian randomization (MR). Summary-level data on 20 serum AAs were obtained from publicly available genome-wide association studies. Genome-wide association studies data on hepatobiliary neoplasms – including primary liver cancer (PLC), hepatocellular carcinoma (HCC), intrahepatic cholangiocarcinoma (ICC), gallbladder and extrahepatic bile duct carcinoma, secondary liver cancer, benign liver tumors, and benign tumors of the extrahepatic bile ducts – were derived from FinnGen and 2 UK Biobank-based studies. A meta-analysis was conducted to calculate pooled effect sizes. Inverse variance weighting was the primary method, supplemented by MR-Egger, weighted median, MR.RAPS, maximum likelihood, and MR-PRESSO methods for sensitive analyses. Higher serum methionine was associated with lower risks of PLC (OR = 0.84, 95% CI: 0.72–0.97, *P* = .021) and HCC (OR = 0.87, 95% CI: 0.80–0.94, *P* < .001), but not ICC. Alanine increased PLC risk (OR = 1.19, 95% CI: 1.00–1.42, *P* = .047), with no significant effect on HCC or ICC. No AAs were linked to gallbladder and extrahepatic bile duct carcinoma or secondary liver cancer. For benign tumors, aspartate (OR = 1.13, 95% CI: 1.01–1.26, *P* = .037), cysteine (OR = 0.72, 95% CI: 0.57–0.92, *P* = .008), and lysine (OR = 1.49, 95% CI: 1.15–1.93, *P* = .003) were significantly associated with benign liver tumors or benign tumors of the extrahepatic bile ducts. This study offers robust evidence of causal associations between specific serum AAs and hepatobiliary neoplasms, emphasizing their potential as biomarkers and modifiable targets for early intervention.

## 1. Introduction

Hepatobiliary neoplasms are the 4th leading cause of cancer-related deaths globally, accounting for approximately 700,000 fatalities each year.^[1,2]^ These neoplasms, encompassing both primary and secondary malignancies, can be further subdivided into more than 20 distinct subtypes.^[3,4]^ Among them, liver cancer ranks as the second most lethal malignancy after pancreatic cancer, with a 5-year survival rate of just 18%.^[5]^ In 2018, approximately 780,000 people died from liver cancer worldwide,^[1]^ and it is expected that annual liver cancer deaths will exceed 1 million by 2030.^[6]^ Hepatocellular carcinoma (HCC), the most common subtype, constitutes over 80% of primary liver cancers (PLC), followed by intrahepatic cholangiocarcinoma (ICC), which accounts for 10% to 15% of cases.^[7]^ Notably, the incidence of ICC has surged by more than 140% over the past few decades.^[8]^ Gallbladder cancer is another highly aggressive malignancy, with approximately 122,000 new cases diagnosed annually. Its global incidence and mortality are increasing, with a 5-year survival rate below 20%.^[9,10]^ Furthermore, a significant proportion of liver cancer cases are metastatic, frequently originating from colorectal cancer.^[11]^ Besides malignant tumors, the hepatobiliary system can develop benign tumors, such as hemangiomas, focal nodular hyperplasia, and hepatocellular adenomas (HCA). While generally nonlife-threatening, these benign tumors may lead to severe complications, including hemorrhage and malignant transformation.^[12]^ Therefore, identifying risk factors for hepatobiliary neoplasms and developing effective intervention strategies are critical for reducing the global disease burden.

The etiology of hepatobiliary neoplasms is complex and multifactorial. HCC is strongly associated with viral hepatitis (HBV and HCV), chronic alcohol consumption, and metabolic syndrome.^[13,14]^ Risk factors for ICC include congenital biliary abnormalities, viral hepatitis, parasitic infections, and diabetes.^[15]^ Gallbladder cancer is linked to multiple causes, including gallstones, aflatoxin exposure, arsenic contamination, obesity, diabetes, and genetic predisposition.^[16]^ In recent years, metabolomics-based research has provided novel insights into the pathogenesis of hepatobiliary neoplasms. Among key metabolites, serum amino acids (AAs) play a critical role in tumor metabolic reprogramming by modulating energy metabolism, biosynthesis, and signaling pathways. Alterations in specific AAs may contribute to the development of hepatobiliary neoplasms, as suggested by previous studies. For instance, Dirk Mossmann et al demonstrated that elevated arginine levels promote liver cancer development.^[17]^ Yang D et al reported an association between elevated branched-chain amino acid levels and an increased risk of liver cancer.^[18]^ Similarly, Ye Y et al found that elevated glutamine levels were significantly associated with a higher risk of HCC.^[19]^ Additionally, Wu T et al and Wei S et al identified elevated serum phenylalanine and valine levels, respectively, in HCC patients.^[20,21]^ However, some studies have reached inconsistent conclusions. For instance, Ma C et al reported no causal relationship between serum AA levels and PLC risk.^[22]^ Similarly, Xu H et al found no causal link between branched-chain amino acid levels and HCC risk.^[23]^ Notably, most previous studies examined serum AA level changes in hepatobiliary tumor patients, reflecting metabolic consequences rather than causal risk factors. Moreover, these studies often suffer from biases and pleiotropic effects, limiting their ability to establish causal relationships. As a robust epidemiological method, Mendelian randomization (MR) effectively overcomes these limitations. By leveraging genetic variants associated with exposures as instrumental variables (IVs), MR enables the estimation of causal effects between exposures and outcomes, minimizing the influence of confounding and reverse causation.^[24]^

This study conducted a comprehensive MR analysis to investigate the causal role of serum AAs in the development of hepatobiliary neoplasms, utilizing the largest and most up-to-date datasets available. The analyses included diverse hepatobiliary tumor types, including PLC, HCC, ICC, gallbladder and extrahepatic bile ducts carcinoma (GB-EBDC), secondary liver cancer (SLC), benign liver tumors (BLT), and benign tumors of extrahepatic bile ducts (BTEBD). Identifying definitive causal associations may provide valuable insights into the potential of serum AAs as biomarkers and therapeutic targets for the hepatobiliary neoplasms.

## 2. Materials and methods

### 2.1. MR design and study samples

**Figure 1.**
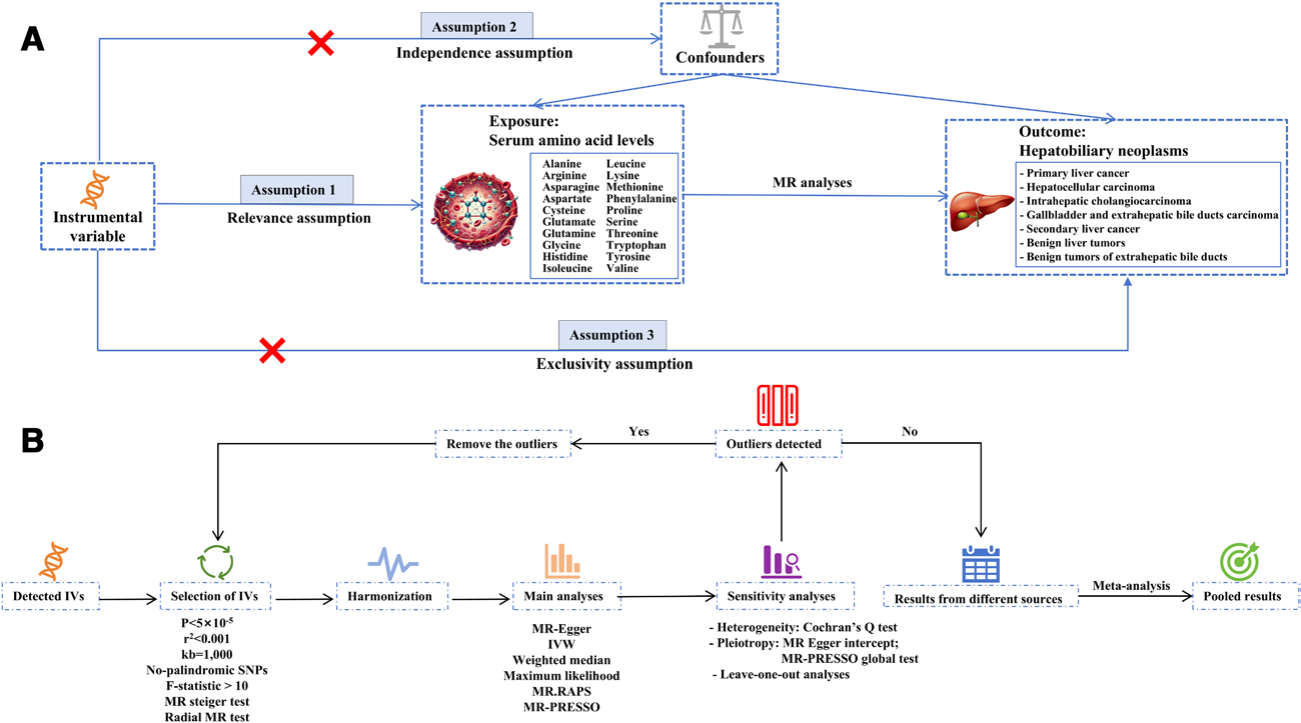
Overview of the MR design and analysis strategy. (A) Overview of the MR design. (B) Analysis strategy of MR. IV = instrumental variables, IVW = inverse variance weighting, MR = Mendelian randomization, MR-PRESSO = MR pleiotropy residual sum and outlier, RAPS = robust adjusted profile score, SNP = single nucleotide polymorphisms.

To investigate the causal relationship between genetically predicted serum AAs and the risk of hepatobiliary neoplasms, we conducted a MR study using summary-level statistics, as shown in Figure 1. The genetic variables must satisfy the 3 core assumptions of MR analysis. First, genetic variables must exhibit a strong association with the exposure of interest (i.e., 20 serum AAs). Second, genetic variables must be independent of confounding factors related to the exposure and outcomes. Third, genetic variables must influence outcomes exclusively through the exposure of interest, minimizing bias from pleiotropy.

The genome-wide association studies (GWAS) datasets for serum AAs were obtained from the Canadian Longitudinal Study of Aging, which included 8299 unrelated participants of European ancestry.^[25]^ Genome-wide genotyping was performed using the Affymetrix Axiom platform, with imputation performed through the Trans-Omics for Precision Medicine program. To ensure data quality, SNPs with an imputation score > 0.3, a minor allele frequency > 0.1%, and a deletion rate < 0.1% were retained. Serum AAs levels were measured using the Metabolon HD4 platform, with values subjected to natural logarithmic transformation and standardization (mean = 0, standard deviation [SD] = 1). Participants with first- or second-degree familial relationships were excluded reduce population stratification bias. Detailed information on datasets of AAs can be found in Table 1 and File S1, Supplemental Digital Content, https://links.lww.com/MD/Q464.

**Table 1:** Detailed description of included GWAS data.

Phenotype	Data source	GWAS_ID	Sample size (case/control)	Descent	PMID
*Exposure*					
Alanine	CLSA	GCST90200431	8265	European	36635386
Arginine	CLSA	GCST90200372	8237	European	36635386
Asparagine	CLSA	GCST90200452	8245	European	36635386
Aspartate	CLSA	GCST90200370	8253	European	36635386
Cysteine	CLSA	GCST90200439	8216	European	36635386
Glutamate	CLSA	GCST90200412	8287	European	36635386
Glutamine	CLSA	GCST90199782	8293	European	36635386
Glycine	CLSA	GCST90200707	8262	European	36635386
Histidine	CLSA	GCST90200377	8223	European	36635386
Isoleucine	CLSA	GCST90200399	8255	European	36635386
Leucine	CLSA	GCST90200389	8252	European	36635386
Lysine	CLSA	GCST90200402	8250	European	36635386
Methionine	CLSA	GCST90200391	8222	European	36635386
Phenylalanine	CLSA	GCST90200396	8228	European	36635386
Proline	CLSA	GCST90200411	8257	European	36635386
Serine	CLSA	GCST90200415	8271	European	36635386
Threonine	CLSA	GCST90200432	8245	European	36635386
Tryptophan	CLSA	GCST90200441	8235	European	36635386
Tyrosine	CLSA	GCST90200427	8252	European	36635386
Valine	CLSA	GCST90200442	8247	European	36635386
*Outcome*					
PLC	FinnGen	/	218,792 (304/218,488)	European	36653562
	Jiang et al (UKB)	GCST90041813	456,348 (214/456,134)	European	34737426
	Zhou et al (UKB)	GCST90435580	393,716 (344/393,372)	European	30104761
HCC	FinnGen	/	345,727 (609/345,118)	European	36653562
	Jiang et al (UKB)	GCST90043858	456,348 (123/456,225)	European	34737426
	Zhou et al (UKB)	GCST90435581	393,513 (141/393,372)	European	30104761
ICC	Jiang et al (UKB)	GCST90043859	456,348 (104/456,244)	European	34737426
GB-EBDC	Jiang et al (UKB)	GCST90041817	456,348 (195/456,153)	European	34737426
	Zhou et al (UKB)	GCST90435586	393,568 (196/393,372)	European	30104761
SLC	Jiang et al (UKB)	GCST90041875	456,348 (835/455,513)	European	34737426
	Zhou et al (UKB)	GSCT90435634	373,242 (2638/370,604)	European	30104761
BLT	FinnGen	/	453,733 (658/453,075)	European	36653562
BTEBD	FinnGen	/	4533,733 (88/453,645)	European	36653562

BLT = benign liver tumors, BTEBD = benign tumors of extrahepatic bile ducts, CLSA = The Canadian Longitudinal Study of Aging, GB-EBDC = gallbladder and extrahepatic bile ducts carcinoma, GWAS = genome-wide association studies, HCC = hepatocellular carcinoma, ICC = intrahepatic cholangiocarcinoma, PLC = primary liver cancer, SLC = secondary liver cancer, UKB = UK Biobank.

For hepatobiliary neoplasms, we utilized distinct datasets for preliminary analysis and external validation, followed by meta-analysis to enhance the robustness of our findings. Summary-level datasets for hepatobiliary neoplasms were obtained from 3 primary sources: First, the FinnGen study, a large-scale genomic initiative analyzing over 500,000 Finnish biobank samples to correlate genetic variations with health data and investigate disease mechanisms.^[26]^ Second, the GWAS conducted by Zhou et al analyzed data from 408,961 European ancestry participants in the UK Biobank.^[27]^ The study employed the SAIGE method with a leave-one-chromosome-out approach, which effectively controls for relatedness and unbalanced case-control ratios, making it well-suited for studying binary traits such as hepatobiliary neoplasms. Third, the GWAS conducted by Jiang et al examined hepatobiliary tumor outcomes in the UK Biobank cohort (n = 456,348) using the fastGWA-GLMM framework.^[28]^ This computationally efficient method uses sparse genomic relationship matrices to adjust for relatedness and incorporates saddlepoint approximation correction to address extreme case-control imbalances, thereby enhancing statistical robustness. These features make this approach especially suitable for analyzing rare variants and complementing methods like SAIGE for detecting genetic associations. Details on datasets of hepatobiliary neoplasms were provided in Files S1 and S2, Supplemental Digital Content, https://links.lww.com/MD/Q464 and https://links.lww.com/MD/Q465.

### 2.2. Selection of IVs

A rigorous screening process was conducted to identify appropriate IVs. SNPs strongly associated with the exposure were identified at a genome-wide significance level of *P* < 5 × 10⁻⁵. Independent SNPs were selected by clumping with a linkage disequilibrium threshold of *r*² < 0.001 within a 1 Mb window, based on the 1000 Genomes Project reference panel. SNPs directly associated with the outcome (*P* < 5 × 10⁻⁵) were excluded to reduce potential bias. Exposure and outcome datasets were harmonized to align effect alleles and exclude palindromic SNPs (File S3, Supplemental Digital Content, https://links.lww.com/MD/Q465). The MR-PRESSO and Radial MR methods were employed to identify and exclude potential outliers, thereby reducing horizontal pleiotropy and enhancing the reliability of causal estimates. The MR-Steiger method was applied to estimate the variance explained by SNPs, confirming that retained SNPs had a stronger association with the exposure than the outcome, thereby mitigating the risk of reverse causality. Weak instrument bias was addressed by calculating the *F* statistic for each SNP using the formula *F* = (Beta/Se)², where Beta denotes the SNP-exposure association coefficient and Se represents the standard error.^[29]^ SNPs with *F* statistics below 10 were excluded. The overall *F* statistic, calculated as the average of individual *F* statistics, exceeded 10, confirming the robustness of the IVs.

### 2.3. Statistical analyses

The primary method for our MR analyses was inverse variance weighting (IVW), which integrates Wald ratios from individual SNP estimates through meta-analysis and assumes all SNPs are valid IVs, enhancing effectively statistical power. To ensure the reliability of our findings, we also used complementary methods such as MR-Egger, weighted median, MR.RAPS, Maximum likelihood, and MR-PRESSO. MR-Egger provides unbiased causal estimates even in the presence of invalid IVs, with its intercept term indicating horizontal pleiotropy.^[30]^ The weighted median provides robust and consistent estimates even when over 50% of SNPs are invalid IVs and demonstrates better control of Type I error rates in small samples compared to the IVW method.^[31]^ MR.RAPS is a statistically robust method that produces reliable results even in the presence of weak instruments or pleiotropy. Maximum likelihood estimation maintains high statistical efficiency, especially in smaller datasets.^[29]^ MR-PRESSO, an IVW variant, identifies and removes outliers to provide robust causal estimates.^[32]^ Heterogeneity and pleiotropy tests were performed to confirm the robustness of our findings. Cochran *Q* test was used to assess heterogeneity, with a *P* value > .05 indicating no significant heterogeneity among the IVs. Additionally, funnel plots were used to visualize heterogeneity. The symmetrical appearance of the plots indicated an even distribution of SNP effect estimates around the overall causal estimate, further supporting the absence of heterogeneity. Horizontal pleiotropy was evaluated using the MR-Egger intercept test and the MR-PRESSO global test, with a *P*-value > .05 indicating no evidence of pleiotropy. Radial MR analysis was conducted to detect potential outlier SNPs, further supporting the absence of pleiotropy.

All statistical analyses were conducted in R (version 4.3.1; R Foundation for Statistical Computing, Vienna, Austria) using packages including “TwoSampleMR,” “tidyverse,” “mr.raps,” “MRPRESSO,” and “RadialMR.”

## 3. Results

### 3.1. Causal effects of AAs on PLC

**Figure 2.**
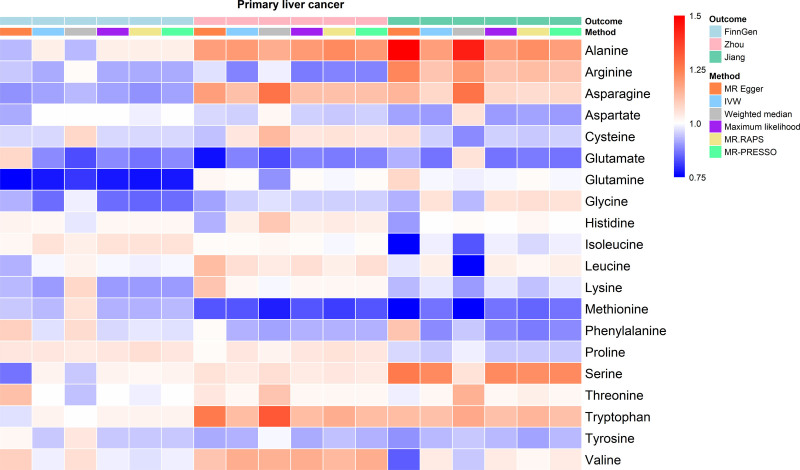
Causal association of serum AAs with PLC among the European population. ORs estimated using IVW method were showed in the heatmap. AAs = amino acids, IVW = inverse variance weighting, MR-PRESSO = MR pleiotropy residual sum and outlier, PLC = primary liver cancer, RAPS = robust adjusted profile score.

**Figure 3.**
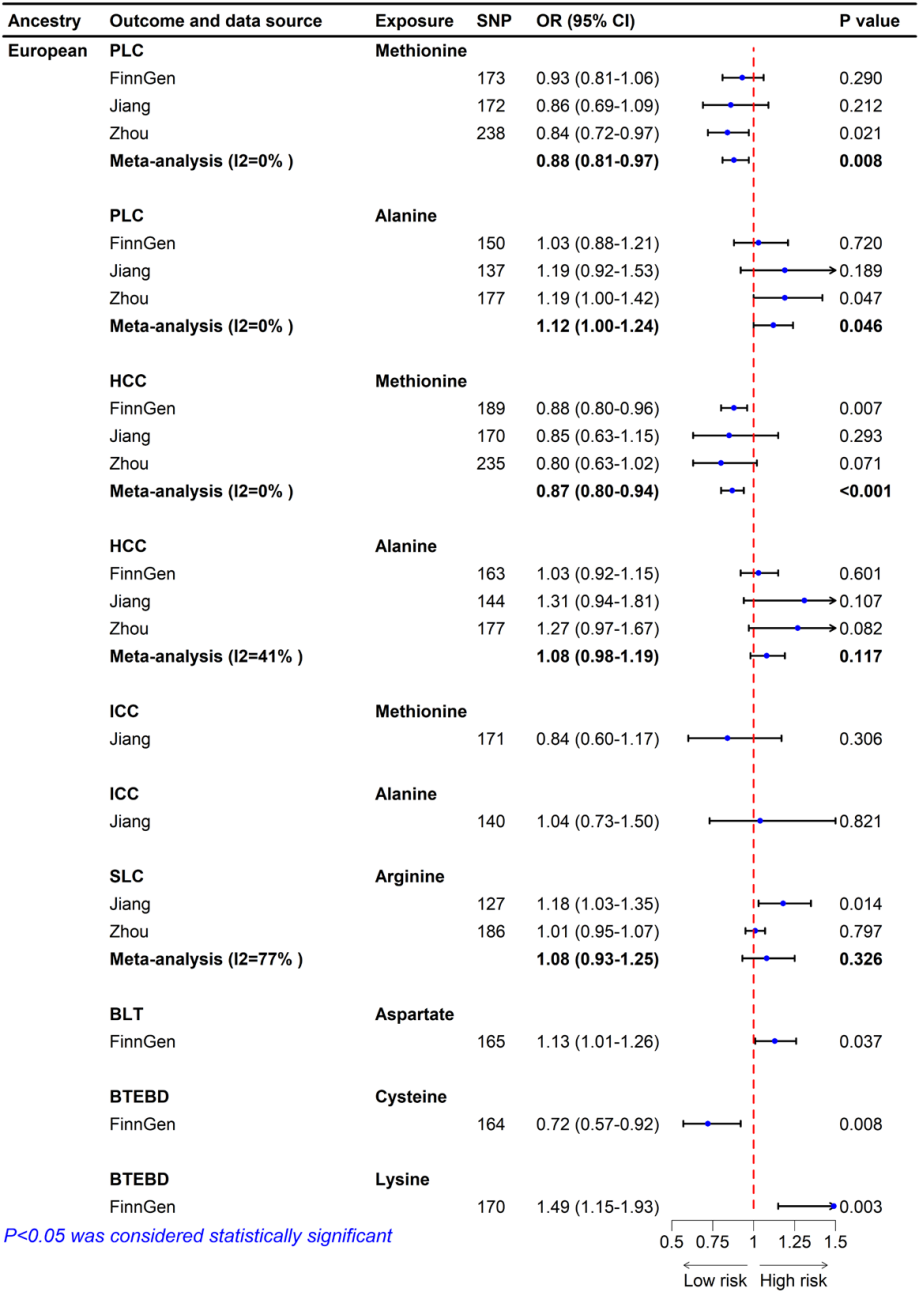
The IVW estimates for the association between serum AAs and hepatobiliary neoplasms. AAs = amino acids, BLT = benign liver tumors, BTEBD = benign tumors of extrahepatic bile ducts, CI = confidence interval, HCC = hepatocellular carcinoma, ICC = intrahepatic carcinoma, IVW = inverse variance weighting, OR = odds ratio, PLC = primary liver cancer, SLC = secondary liver cancer, SNP = single nucleotide polymorphisms.

**Figure 4.**
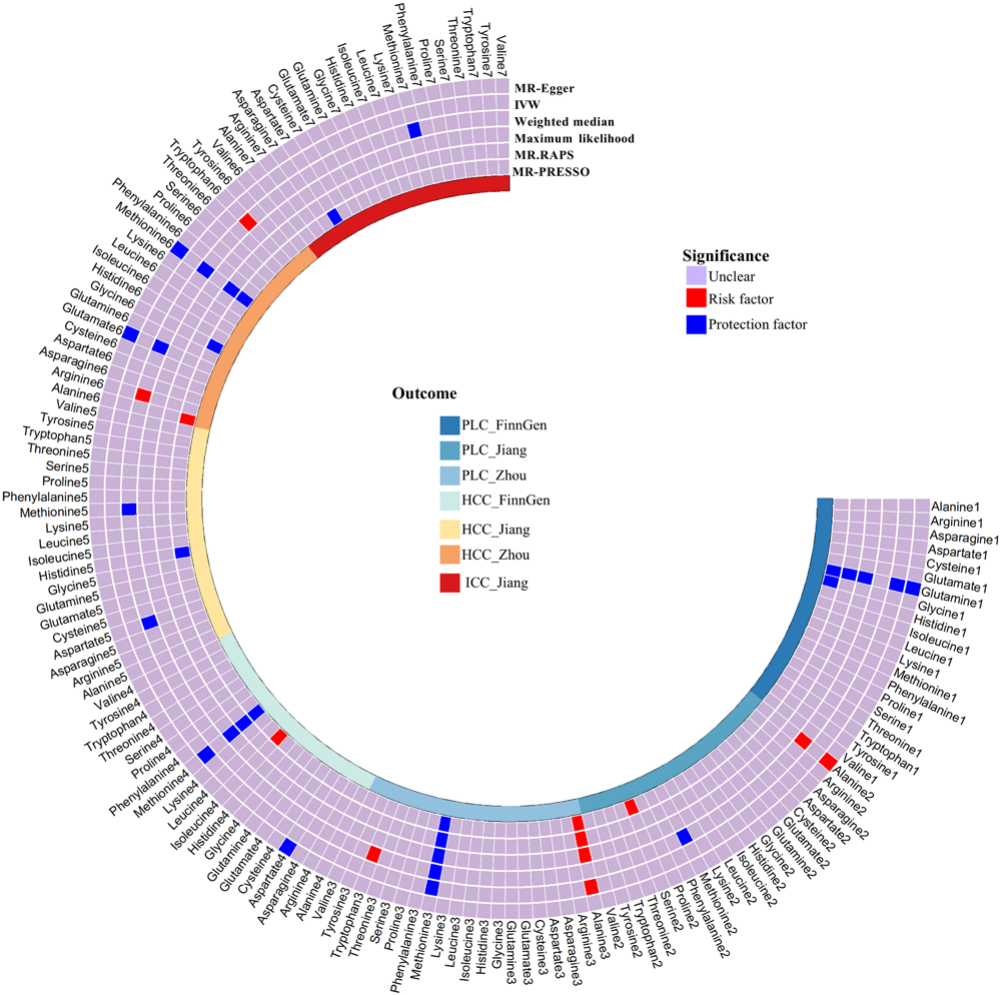
MR analyses of serum AAs on PLC, HCC, and ICC in the discovery and replication GWASs. The MR analyses were performed and the results of 6 approaches including IVW, MR-Egger, Weighted median, Maximum likelihood, MR.RAPS, and MR-PRESSO were summarized here. AAs = amino acids, GWASs = genome-wide association studies, HCC = hepatocellular carcinoma, ICC = intrahepatic carcinoma, IVW = inverse variance weighting, MR-PRESSO = MR pleiotropy residual sum and outlier, PLC = primary liver cancer, RAPS = robust adjusted profile score.

The causal relationship between 20 serum AAs and PLC are shown in Figure 2. Specifically, based on GWAS data from Zhou et al, genetically predicted methionine levels were significantly inversely associated with PLC risk (IVW, OR = 0.84, 95% CI: 0.72–0.97, *P* = .021), while alanine levels were positively associated with PLC risk (IVW, OR = 1.19, 95% CI: 1.00–1.42, *P* = .047). The direction of these associations was consistent in the FinnGen database and the GWAS data from Jiang et al (Fig. 3). Meta-analysis of the 3 data sources showed that each SD increase in genetically predicted methionine levels reduced PLC risk to 0.88 (95% CI: 0.81–0.97, *P* = .008). Each SD increase in genetically predicted alanine levels was associated with a 1.12-fold increase in PLC risk (95% CI: 1.00–1.24, *P* = .046). Except for the Weighted Median method in the FinnGen data, all sensitivity analyses yielded consistent causal association. Additionally, FinnGen data identified a significant inverse association between serum glutamine levels and PLC risk (IVW, OR = 0.77, 95% CI: 0.65–0.92, *P* = .004). These findings were further validated by MR-Egger (OR = 0.76, 95% CI: 0.60–0.95, *P* = .02), Maximum Likelihood (OR = 0.77, 95% CI: 0.65–0.92, *P* = .004), MR-RAPS (OR = 0.77, 95% CI: 0.64–0.92, *P* = .005), and MR-PRESSO (OR = 0.77, 95% CI: 0.67–0.89, *P* < .001; Fig. 4 and File S4, Supplemental Digital Content, https://links.lww.com/MD/Q465). However, GWAS data from Zhou et al showed a positive association direction between serum glutamine levels and PLC risk (OR = 1.01, 95% CI: 0.83–1.22, *P* = .96), indicating that further research is needed to establish a definitive causal relationship. Scatter plots illustrate the direction of causal relationships between serum AAs and PLC (File S5, Supplemental Digital Content, https://links.lww.com/MD/Q464). No heterogeneity was detected in Cochran *Q* test or funnel plots with symmetrical distribution (File S6, Supplemental Digital Content, https://links.lww.com/MD/Q464). Neither the MR-Egger intercept test nor the MR-PRESSO global test identified pleiotropy (*P* > .05, File S7, Supplemental Digital Content, https://links.lww.com/MD/Q465). In addition, the leave-one-out analyses based on the IVW method did not identify SNPs with significant impacts on the results, further supporting the robustness of the findings (File S8, Supplemental Digital Content, https://links.lww.com/MD/Q464).

### 3.2. Causal effects of AAs on HCC

**Figure 5.**
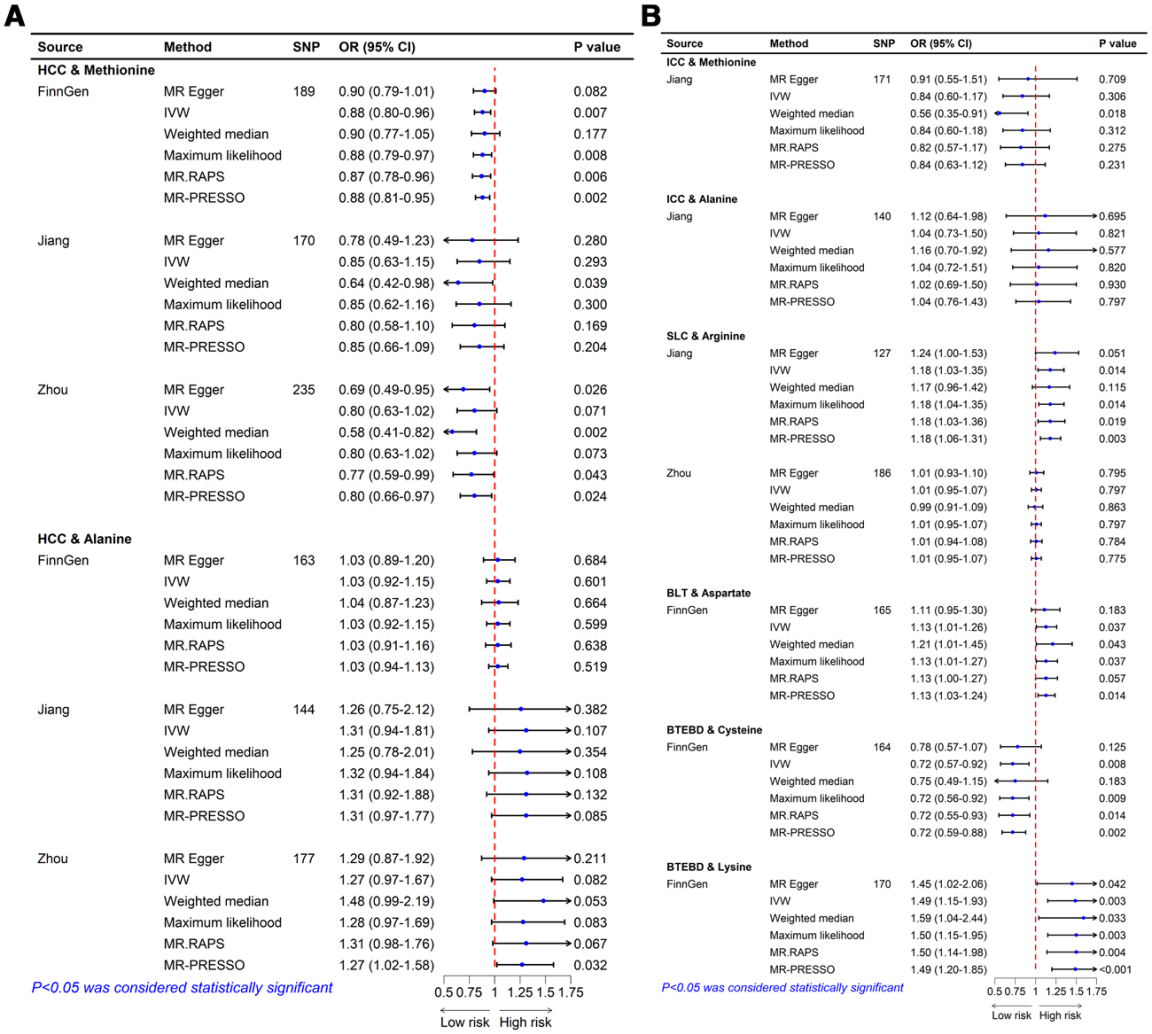
Causal association of serum AAs with HCC (A), ICC, SLC, BLT and BTEBD (B). AAs = amino acids, BLT = benign liver tumors, BTEBD = benign tumors of extrahepatic bile ducts, CI = confidence interval, HCC = hepatocellular carcinoma, ICC = intrahepatic carcinoma, IVW = inverse variance weighting, MR-PRESSO = MR pleiotropy residual sum and outlier, OR = odds ratio, RAPS = robust adjusted profile score, SLC = secondary liver cancer, SNP = single nucleotide polymorphisms.

Based on the FinnGen database, there is significant evidence that genetically predicted serum methionine levels reduce HCC risk (IVW, OR = 0.88, 95% CI: 0.80–0.96, *P* = .007). The direction of this association was consistent with the GWAS data from Zhou et al and Jiang et al (Fig. 3). Meta-analysis of the 3 data sources estimated that each SD increase in genetically predicted methionine level reduced HCC risk to 0.87 (95% CI: 0.80–0.94, *P* < .001). No significant evidence supported a causal association between genetically predicted alanine levels and HCC risk (IVW, OR = 1.08, 95% CI: 0.98–1.19, *P* = .117). All sensitivity analyses yielded consistent causal association (Fig. 5A). No evidence of heterogeneity and horizontal pleiotropy was detected in the IVs (File S7, Supplemental Digital Content, https://links.lww.com/MD/Q465).

### 3.3. Causal effects of AAs on ICC

Due to limited ICC data sources, we explored the causal relationship between AAs and ICC using only Jiang et al GWAS. The results indicated that the direction of causal association between genetically predicted methionine or alanine levels and the risk of ICC was consistent with HCC, while the estimates were not statistically significant (both *P* > .05). Causal associations remained consistent across all sensitivity analyses (Fig. 5B), supporting the robustness of the results. No evidence of heterogeneity or horizontal pleiotropy was found (File S7, Supplemental Digital Content, https://links.lww.com/MD/Q465). The leave-one-out plot revealed no SNPs with a significant effect on the results (File S8, Supplemental Digital Content, https://links.lww.com/MD/Q464).

### 3.4. Causal effects of AA on GB-EBDC

Due to the limited data sources on GB-EBDC, we only explored the causal correlation between AAs and the risk of GB-EBDC based on Jiang et al GWAS and Zhou et al GWAS. No significant causal associations were identified between any serum AAs and the risks of GB-EBDC. Consistent causal associations were found across all sensitivity analyses. File S7, Supplemental Digital Content, https://links.lww.com/MD/Q465 confirmed no evidence of heterogeneity or horizontal pleiotropy (*P* > .05).

### 3.5. Causal effects of AA on SLC

Based on Jiang et al GWAS, genetically predicted serum arginine was significantly positively associated with the risk of SLC (OR = 1.18, 95% CI = 1.03–1.35, *P* = .014), consistent with the causal inference from Zhou et al GWAS (Fig. 3), although the latter did not reach statistical significance (OR = 1.01, 95% CI = 0.95–1.07, *P* = .797). As Figure 5B shown, consistent causal associations were found in sensitivity analyses such as MR-Egger, Maximum likelihood, MR.RAPS and MR-PRESSO. However, a meta-analysis combining the results from both studies showed no statistically significant association (OR = 1.08, 95% CI = 0.93–1.25, *P* = .326), although the OR value suggested a potential increased risk of SLC with higher serum arginine levels. Therefore, further studies using larger and more comprehensive datasets are needed in the future.

### 3.6. Causal effects of AA on BLT and BTEBD

Based on the FinnGen dataset, genetically predicted serum aspartate were positively associated with the risk of BLT (IVW, OR = 1.13, 95% CI: 1.01–1.26, *P* = .037). Serum cysteine was negatively associated with the risk of BTEBD (IVW, OR = 0.72, 95% CI = 0.57–0.92, *P* = .008), while serum lysine was positively associated (IVW, OR = 1.49, 95% CI = 1.15–1.93, *P* = .003; Fig. 3). Sensitivity analyses, including MR-Egger, Weighted Median, MR-RAPS, Maximum Likelihood, and MR-PRESSO, confirmed consistent causal associations (Fig. 5B). The MR-Egger intercept and MR-PRESSO global test showed no evidence of horizontal pleiotropy, while the symmetrical funnel plot and Cochran *Q* test indicated no heterogeneity.

## 4. Discussion

In this MR study, we systematically investigated the causal relationships between serum AAs and hepatobiliary neoplasms. The results showed that genetically predicted serum methionine significantly reduced the risk of PLC and HCC, while serum alanine increased the risk of PLC. Serum aspartate elevated the risk of BLT, serum lysine increased the risk of BTEBD, and serum cysteine reduced the risk of the latter. These findings suggest that specific serum AAs may serve as biomarkers or therapeutic targets for hepatobiliary neoplasms, and early identification and management of AAs imbalances could help reduce the incidence of these diseases. Additionally, the complex and occasionally inconsistent associations between serum AAs and tumor types highlight the need for mechanistic insights to elucidate these relationships.

Our analysis revealed that genetically predicted serum methionine significantly reduced the risk of PLC and HCC, but showed no significant protective effect against ICC. This discrepancy may arise from fundamental differences in the biological and metabolic characteristics of HCC and ICC. Methionine is converted to S-adenosylmethionine (SAM) by methionine adenosyltransferase.^[33]^ SAM acts as a key methyl donor for DNA and histone methylation, playing a crucial role in regulating hepatocyte proliferation, differentiation, and apoptosis.^[34–36]^ Reduced SAM levels are linked to DNA hypomethylation, genomic instability, and oxidative stress, all of which contribute to hepatocarcinogenesis.^[34,37]^ SAM also enhances antioxidant defense by promoting glutathione (GSH) synthesis and reduces liver inflammation through TNF-α inhibition, thereby preventing HCC development.^[38,39]^ Pascale et al showed that increasing SAM levels in rats inhibited the progression of persistent hepatic nodules and HCC.^[40]^ Other studies have also reported the chemopreventive effects of SAM against experimental liver cancer in humans.^[41]^ In contrast, ICC pathogenesis mainly involves bile duct inflammation and cholestasis, driven by changes in the biliary microenvironment rather than epigenetic dysregulation. Moreover, while SAM’s anti-inflammatory effects via TNF-α suppression are significant in hepatic inflammation, ICC-related biliary inflammation is predominantly mediated by cytokines such as interleukin-6.^[42]^ These distinctive biological features likely limit the potential preventive role of methionine and its metabolites in ICC.

Few studies have explored the causal association between serum alanine and hepatobiliary neoplasms. Ning et al reported significantly elevated serum alanine levels in patients with HCC.^[43]^ Our results indicated that serum alanine levels were significantly associated with an increased risk of PLC. However, no significant causal relationship was observed for HCC or ICC when analyzed as separate subtypes, despite OR values exceeding 1. Alanine, a key component of the glucose-alanine cycle, promotes glucose production and maintains energy supply. Elevated serum alanine levels may accelerate tumor cell energy metabolism by enhancing the Warburg effect, thereby contributing to carcinogenesis.^[44]^ Additionally, as a precursor for pyruvate and acetyl-CoA, alanine supports the TCA cycle and increases ROS production, potentially leading to oxidative stress and genomic instability. However, the metabolic demands of HCC and ICC involve more complex and subtype-specific pathways, potentially attenuating alanine’s independent effects. Furthermore, the limited study sample size may reduce statistical power, contributing to the lack of significance for individual subtypes.^[45]^ In summary, elevated plasma alanine levels may increase PLC risk via metabolic dysregulation and oxidative stress. The nonsignificant findings for HCC and ICC highlight the complexity of alanine’s role in liver cancer subtypes, necessitating further research with metabolomic profiling, experimental validation, and larger datasets to clarify underlying mechanisms.

In this study, we observed a significant positive association between genetically predicted serum arginine levels and the risk of SLC based on Jiang et al GWAS. However, no significant association was found in Zhou et al GWAS, despite an OR > 1. The meta-analysis combining both datasets also yielded nonsignificant results (OR > 1). Arginine, a key component of the urea cycle and nitric oxide synthesis, contributes to tumor progression via metabolic and transcriptional regulation.^[46]^ For example, Prasad YR et al demonstrated that elevated arginine levels promote cancer by upregulating metabolic genes, such as asparagine synthase.^[47]^ Additionally, the pro-tumor effects of arginine may depend on the specific tumor microenvironment of liver metastases, where it can enhance angiogenesis and support tumor cell proliferation.^[48]^ The lack of statistical significance in the meta-analysis suggests that the observed association may be affected by insufficient statistical power, emphasizing the need for larger, well-characterized cohorts to validate arginine’s causal role in SLC and clarify underlying biological mechanisms.

For benign tumors, serum aspartate was positively associated with the risk of BLT, which may be related to its key role in TCA cycle. Aspartate can enhance metabolic activity, promoting abnormal cell proliferation and tumor growth. Elevated serum aspartic acid levels are associated with increased risks of diabetes, prostate cancer, and thyroid cancer,^[49–51]^ indirectly supporting the potential mechanism that promotes cell proliferation. Serum cysteine levels were inversely associated with the risk of BTEBD, likely due to its role as a precursor of GSH. By enhancing antioxidant defense and reducing oxidative stress in the biliary epithelium, cysteine potentially mitigates damage that could lead to tumorigenesis.^[52]^ Additionally, cysteine contributes to immune regulation by maintaining immune homeostasis, thereby reducing the tumor risk.^[53]^ Conversely, serum lysine levels were positively associated with the risk of BTEBD, possibly due to its role in promoting cell growth and proliferation through the mammalian target of rapamycin signaling pathway. Mammalian target of rapamycin is a core pathway that regulates cell proliferation, and its overactivation may promote tumorigenesis.^[54]^ Zhang R et al demonstrated that lysine deprivation induces cell cycle arrest and apoptosis in HCC cell lines,^[55]^ supporting the critical role of lysine in cell survival. These findings underscore the diverse metabolic roles of AAs in benign hepatobiliary tumor development and highlight the need for further research to clarify the molecular mechanisms underlying these associations.

This MR study has several significant advantages. Specifically, first, this is the MR study to systematically explore the causal relationship between serum AAs levels and hepatobiliary neoplasms, including both malignant and benign types. Second, this study utilizes 3 large, high-quality GWAS datasets: FinnGen, Jiang et al GWAS, and Zhou et al GWAS, enabling validation of findings across multiple sources. Additional meta-analysis further reinforces the robustness of the findings. Third, the study was limited to populations of European descent, reducing potential bias from demographic stratification. Fourth, the MR approach effectively overcomes inherent limitations of observational studies, such as confounding bias and reverse causality, minimizing bias in causal estimates under specific assumptions. Finally, strict IV screening criteria were applied, and a series of sensitivity analyses, including MR-Egger, MR-PRESSO, and leave-one-out analysis, confirmed the robustness and consistency of the results. However, this MR study also has several limitations. First, restriction to populations of European ancestry limits the generalizability of the findings to other populations. Second, certain subtypes, such as ICC and SLC, had relatively small sample sizes, potentially limiting the ability to detect significant associations. Furthermore, while sensitivity analysis confirmed the robustness of our findings, some inconsistent results between different datasets highlighted the potential impact of dataset specific factors, underscoring the need for larger, more comprehensive datasets in future studies. Third, the lack of individual-level data prevents stratify analyses based on potential influencing factors such as sex, age, or specific comorbidities. Fourth, although our study identified a significant causal relationship, the underlying molecular mechanisms are still not fully understood. Further functional studies are needed to elucidate the biological pathways of serum AAs and hepatobiliary tumorigenesis.

## 5. Conclusion

This MR study systematically explored the causal relationships between AAs and hepatobiliary neoplasms, revealing novel metabolic insights. Higher serum methionine levels were associated with a reduced risk of PLC and HCC, whereas elevated alanine increased PLC risk. Additionally, serum aspartate was linked to a higher risk of BLT, while serum lysine increased and serum cysteine decreased the risk of BTEBD. These findings underscore the potential role of AAs metabolism in hepatobiliary tumorigenesis and highlight the need for early metabolic monitoring and targeted interventions for risk mitigation.

## Acknowledgments

The authors sincerely thank the referenced studies or consortiums for contributing open-access datasets for the analysis.

## Author contributions

**Conceptualization:** Aoqiang Zhai, Li Ren, Haijie Hu, Fuyu Li.

**Data curation:** Aoqiang Zhai, Li Ren.

**Formal analysis:** Ruiqi Zou, Siqi Yang.

**Funding acquisition:** Yanwen Jin, Haijie Hu, Fuyu Li.

**Investigation:** Yanwen Jin.

**Methodology:** Aoqiang Zhai, Li Ren.

**Supervision:** Haijie Hu, Fuyu Li.

**Validation:** Aoqiang Zhai, Li Ren, Yanjie Zhong.

**Visualization:** Aoqiang Zhai.

**Writing – original draft:** Aoqiang Zhai.

**Writing – review & editing:** Haijie Hu, Fuyu Li.

## Supplementary Material


