# Supplementary material for: Unveiling causal links between serum amino acid levels and risk of hepatobiliary neoplasms by Mendelian randomization study
Source: Medicine (Baltimore). 2025 Oct 24;104(43):e45491. doi: 10.1097/MD.0000000000045491 (PMC12558176; doi:10.1097/MD.0000000000045491)
Supplement: Supplementary file 1 [file medi-104-e45491-s001.docx]

**Supplementary File S1:** Details of included GWASs. The detailed information regarding the phenotype, quality control process, etc., is displayed here.

**Detailed information of 20 serum amino acids (AAs)**

The serum AAs included in the study included Alanine, Arginine, Asparagine, Aspartate, Cysteine, Glutamate, Glutamine, Glycine, Histidine, Isoleucine, Leucine, Lysine, Methionine, Phenylalanine, Proline, Serine, Threonine, Tryptophan, Tyrosine, and Valine. The GWAS data for serum amino acids were derived from the Canadian Longitudinal Study of Aging (CLSA), including 8,299 unrelated European individuals. To minimize population stratification bias, non-European individuals and those with first- or second-degree relatives (n = 203) were excluded using kinship-based inference. Genotyping was performed using the Affymetrix Axiom platform, followed by imputation with the TOPMed reference panel. SNPs were filtered based on MAF > 0.1%, imputation quality > 0.3, and missing rate < 0.1, yielding approximately 15.4 million SNPs (build 38). Plasma metabolites were quantified using the Metabolon HD4 platform (UPLC-MS/MS). Strict quality control ensured accurate metabolite identification, retaining only those with <50% missing values (N = 1,091). Metabolite levels were log-transformed, outliers (±3 SD) removed, and values standardized. GWAS was conducted using fastGWA in GCTA (v1.93.2 beta), adjusting for age, sex, fasting status, genotyping batch, and the first 10 genetic principal components.

**Detailed information of hepatobiliary neoplasms**

The hepatobiliary neoplasms included in the study included primary liver cancer (PLC), hepatocellular carcinoma (HCC), intrahepatic cholangiocarcinoma (ICC), gallbladder and extrahepatic bile ducts [carcinoma](https://www.ebi.ac.uk/gwas/efotraits/MONDO_0018918) (GB-EBDC), secondary liver cancer (SLC), benign liver tumors (BLT), and benign tumors of extrahepatic bile ducts (BTEBD). The summary-level datasets were derived from three GWAS datasets: FinnGen database and two UK Biobank-based studies by Jiang et al. and Zhou et al.

The FinnGen study is a large-scale genomics initiative that has analyzed over 500,000 Finnish biobank samples and correlated genetic variation with health data to understand disease mechanisms and predispositions. Phenotypes, referred to as "end points," were defined by integrating data from multiple nationwide health registers. The study initially constructed over 2,800 end points by combining data from hospital discharge records, prescription medication purchase records, cancer registries, and other national health databases. In the GWAS of hepatobiliary neoplasms, FinnGen leveraged this comprehensive health registry-based phenotype classification, enabling a large-scale genetic investigation of disease risk. Samples were genotyped with Illumina (Illumina) and Affymetrix arrays (Thermo Fisher Scientific). The mixed-model logistic regression method SAIGE (v.0.35.8.8) was used for association analysis. Sex, age, genotyping batch, and ten PCs were used as covariates.

Zhou et al. conducted a GWAS on hepatobiliary neoplasms using SAIGE, a scalable mixed model method that corrects type I error inflation for low-frequency variants in binary traits. SAIGE employs saddlepoint approximation for accurate p-values, even with unbalanced case-control ratios, and optimizes computation for large biobank-scale studies. Their analysis included 408,961 White British UK Biobank participants, examining 28 million genetic markers across 1,403 binary traits. The logistic mixed model incorporated sex, birth year, and the first four principal components as covariates. Phenotypes were defined by mapping International Classification of Disease (ICD)-10 codes to PheCodes using UMLS resources, string matching, and manual review. Cases included individuals with the PheCode of interest, while controls were those without it or related codes. Gender-specific PheCodes were verified to prevent misclassification.

Jiang et al. conducted a GWAS on hepatobiliary neoplasms using fastGWA-GLMM, a highly efficient GLMM-based tool optimized for large-scale biobank data. By leveraging sparse matrix algorithms, fastGWA-GLMM achieves significantly higher computational efficiency than existing methods, making it scalable to cohorts with millions of individuals. Simulation studies confirmed that its test statistics for both common and rare variants remain well-calibrated under the null, even for traits with extreme case-control imbalances. Their analysis included 456,348 UK Biobank participants, examining 11,842,647 variants across 2,989 binary traits. Genotyping was performed using two arrays: the UK Biobank Axiom Array and the UK BiLEVE Axiom Array. SNP imputation was conducted by the UK Biobank team using reference panels from the Haplotype Reference Consortium and the UK10K project, with standard quality control thresholds applied. The GWAS model accounted for age, sex, their interaction terms, and the top 20 principal components. The phenotypes were generated using the pipeline from the Neale Lab (http://www.nealelab.is/uk-biobank) or their in-house pipeline based on the ICD to Phecode maps. Cases included individuals with the PheCode of interest, while controls were those without the code or related conditions.

For phenotype definition of the relevant outcome variables, please see Supplementary File S2.

**Supplementary File S5:** Scatter plot of the 20 serum AAs in the discovery and replication of hepatobiliary neoplasms GWASs.


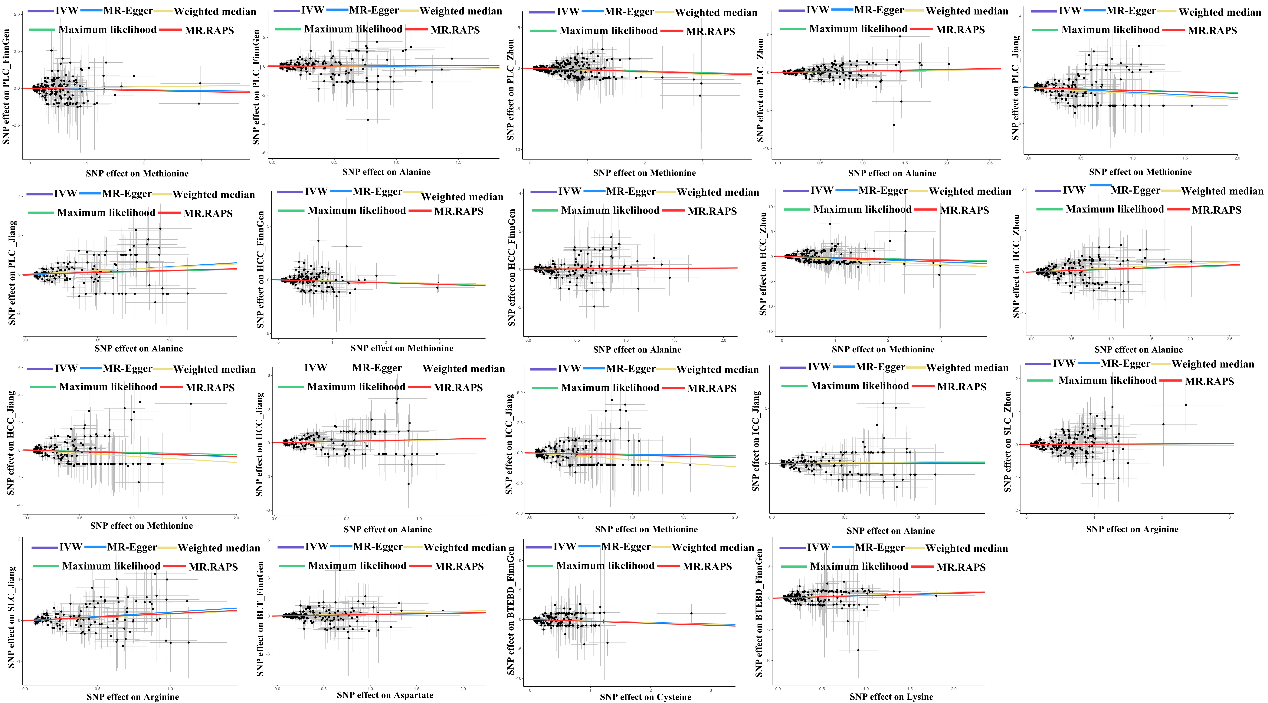


AAs: amino acids; GWASs: genome-wide association studies.

**Supplementary File S6：**Funnel plot of the 20 serum AAs in the discovery and replication of hepatobiliary neoplasms GWASs.


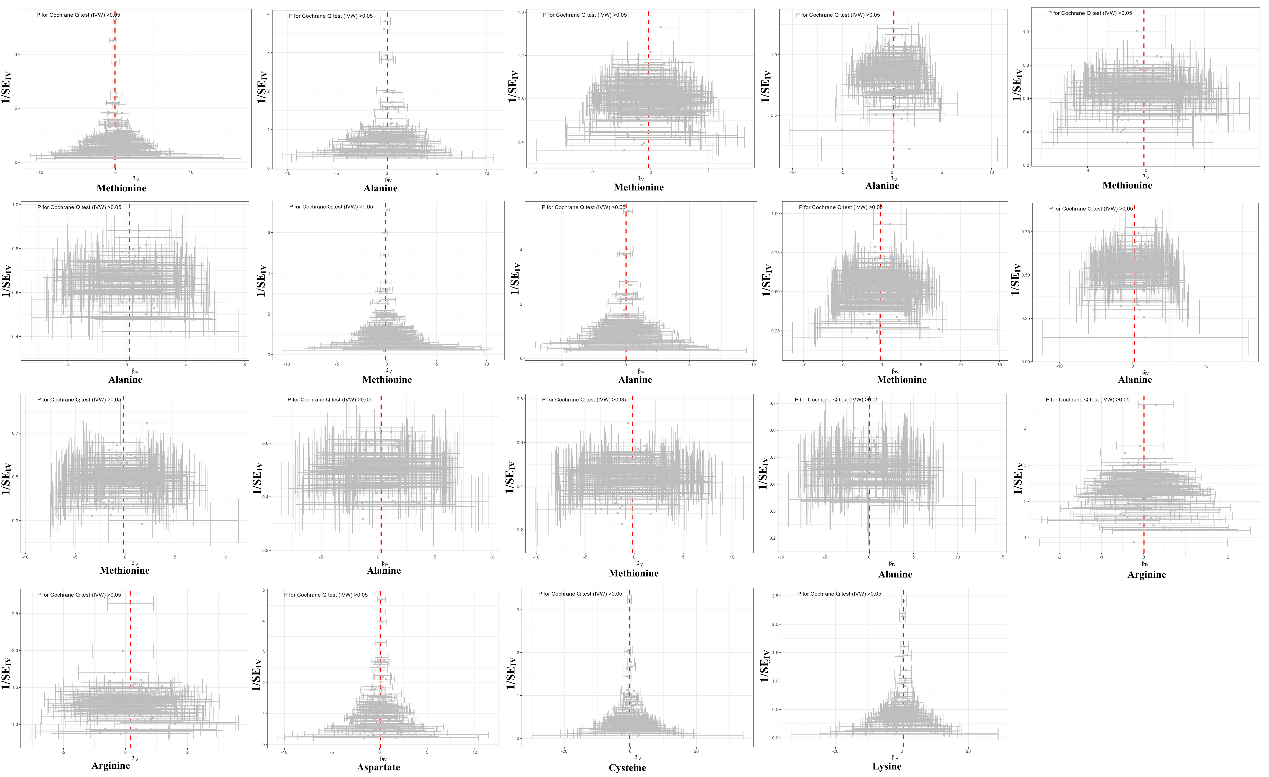


The heterogeneity of 20 serum AAs is quantified using Cochran’s Q test, and then visualized using funnel plot. AAs: amino acids; GWASs: genome-wide association studies.

**Supplementary File S8.** Leave-one-out plot of the 20 serum AAs in the discovery and replication of hepatobiliary neoplasms GWASs.


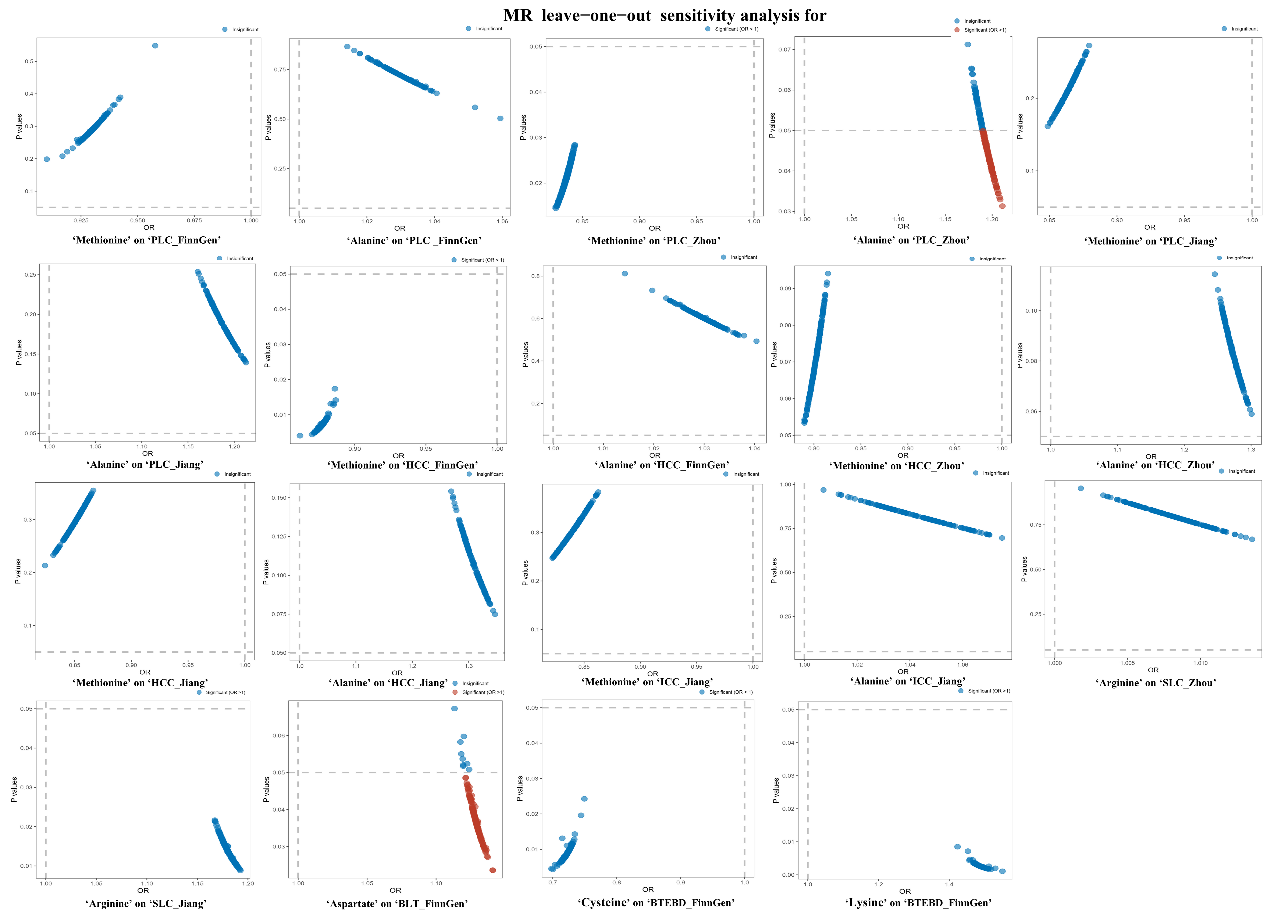


The basic leave-one-out plot was processed by volcano mapping, with p value as the ordinate and OR as the horizontal coordinate. Significant (OR>1): P < 0.05 and OR > 1; Significant (OR < 1): P < 0.05 and OR < 1; Insignificant: P > 0.05. AAs: amino acids; GWASs: genome-wide association studies.
